# Mapping Soil Alkalinity and Salinity in Northern Songnen Plain, China with the HJ-1 Hyperspectral Imager Data and Partial Least Squares Regression

**DOI:** 10.3390/s18113855

**Published:** 2018-11-09

**Authors:** Lin Bai, Cuizhen Wang, Shuying Zang, Changshan Wu, Jinming Luo, Yuexiang Wu

**Affiliations:** 1 Heilongjiang Province Key Laboratory of Geographical Environment Monitoring and Spatial Information Service in Cold Regions, Harbin Normal University, Harbin 150025, China; bail986@163.com; 2 Department of Science, Qiqihar University, Qiqihar 161006, China; Luojm1000@163.com; 3 Department of Geography, University of South Carolina, Columbia, SC 29208, USA; CWANG@mailbox.sc.edu; 4 Department of Geography, University of Wisconsin-Milwaukee, P.O. Box 413, Milwaukee, WI 53201, USA; cswu@uwm.edu; 5 Qiqihar Meteorological Bureau, Qiqihar 161006, China; qxjwyx@163.com

**Keywords:** soil, alkalinity and salinity, hyperspectral data, PLSR model

## Abstract

In arid and semi-arid regions, identifying and monitoring of soil alkalinity and salinity are in urgently need for preventing land degradation and maintaining ecological balances. In this study, physicochemical, statistical, and spectral analysis revealed that potential of hydrogen (pH) and electrical conductivity (EC) characterized the saline-alkali soils and were sensitive to the visible and near infrared (VIS-NIR) wavelengths. On the basis of soil pH, EC, and spectral data, the partial least squares regression (PLSR) models for estimating soil alkalinity and salinity were constructed. The R^2^ values for soil pH and EC models were 0.77 and 0.48, and the root mean square errors (RMSEs) were 0.95 and 17.92 dS/m, respectively. The ratios of performance to inter-quartile distance (RPIQ) for the soil pH and EC models were 3.84 and 0.14, respectively, indicating that the soil pH model performed well but the soil EC model was not considerably reliable. With the validation dataset, the RMSEs of the two models were 1.06 and 18.92 dS/m. With the PLSR models applied to hyperspectral data acquired from the hyperspectral imager (HSI) onboard the HJ-1A satellite (launched in 2008 by China), the soil alkalinity and salinity distributions were mapped in the study area, and were validated with RMSEs of 1.09 and 17.30 dS/m, respectively. These findings revealed that the hyperspectral images in the VIS-NIR wavelengths had the potential to map soil alkalinity and salinity in the Songnen Plain, China.

## 1. Introduction

Soil alkalinity and salinity are the primary causes of land desertification, which usually occurs in irrigated farmland and grassland in arid and semiarid regions. As a severe environmental hazard, soil alkalinization and salinization negatively affects crop yields and agricultural productivity [1], thereby posing serious threats to ecosystem health and economic sustainability. Soils in dry lands worldwide, including those in the United States, China, Hungary, and Australia, suffer desertification and land degradation [2]. The total area of saline soil in China is 3.6 × 10^7^ ha [3], approximately 9% of which is in the Songnen Plain [4]. Located in the central part of northeastern China, the Songnen Plain is composed of saline-alkali soils that are mainly due to soda [5]. The plain has wide distributions of igneous alkaline rocks that contain abundant sodium aluminosilicates, such as orthoclase, plagioclase, albite, sodalite, and nepheline [6]. These igneous alkaline rocks, which serve as parent materials of soil, are closely related to soil salinization and alkalinization in the Songnen Plain [4,6]. Inside the plain, the land is flat and the slope is gentle, the streams are slow running, the drainage is blocked, and salts are difficult to leach out [7]. Such geomorphic pattern is shaped by Neotectonic movement and favors soil salinization and alkalinization [6]. So, most salt-affected areas correspond to lacustrine depressions, especially in areas where temporary water logging occurs due to insufficient natural drainage [4]. Soil alkalinity and salinity are affected by the monsoon climate greatly. The annual evaporation is larger than precipitation and the humidity is low in Songnen Plain [4,8]. Salts are accumulated in soil surface by strong evaporation in dry season, whereas they are transported from soil surface to soil bottom by precipitation in the rainy season. As a result, the potential of hydrogen (pH) and electrical conductivity (EC) of the soil surface might be strongly affected by seasonality. Moreover, freeze-thawing process favors soil salinization and alkalinization in the plain [4,8,9]. In addition to geomorphic reasons, the human-related factors, such as overgrazing, fertilizers and unreasonable utilization of land and water accelerate the process of soil salinization and alkalinization [4].

Remote sensing technology obtains substantial soil information in large spatial coverage, which could be superior to traditional field surveys that are expensive and time consuming. For this reason, remote sensing data have been applied to monitor saline-alkali soils for decades. A variety of remote sensing data, such as aerial photographs, video images, infrared thermography, multispectral images and microwave images, have been used to identify and monitor salt-affected soils [1]. Multispectral imagery was used to detect different levels of soil alkalinity and salinity with visual interpretation of false-color composites [10,11]. However, this analytical interpretation is subjective and time consuming. Metternicht [12] applied fuzzy logic with Landsat Thematic Mapper (TM) images to detect salt types in a salt-affected area of Bolivia. Although numerous multispectral sensors have been successful in distinguishing severely salt-affected soils from non-affected soils [13], they were reported to have limitations in quantitatively estimating soil properties due to their low spectral resolution and the use of conventional classification methods [14]. Some attempts began to estimate soil properties by using multispectral images together with field spectroradiometer data. Bannari et al. [15] constructed an index based on the optimal Advanced Land Imaging (ALI) bands with spectral data to estimate soil EC and delineate slight and moderate saline and sodic soil. Bai et al. [16] used stepwise regression based on the optimal Landsat 8 Operational Land Imager (OLI) bands with spectrometer data to estimate soil pH and EC quantitatively in a typical saline-alkali area in Northeast China. However, the broad band of these images may smooth out the essential but subtle characteristics in soil spectra, resulting in the inefficiency or inefficacy of the estimation from the multispectral imagery. 

Hyperspectral remote sensing overcomes the bandwidth restriction of multispectral imagery. The predictive model or indexes based on its narrow and spectrally continuous bands may detect the subtle variations in soil characteristics. Numerous studies have detected the spectral characteristics of materials [17]. McCarty et al. [18] and McCarty and Reeves [19] found that mid-infrared spectroscopy was optimal for estimating soil carbon content, total nitrogen, and soil pH. Vohland et al. [20] discovered that the key wavelengths of soil pH were restricted to the spectral regions beyond 1915 nm in carbonate land. Hunt and Salisbury [21] found that ${\mathrm{CO}}_{3}^{2-}$ had the clearest absorption at 2300–2350 nm. Hyperspectral remote sensing provides near-laboratory reflectance spectra for each pixel [22] and thus has the potential to monitor saline-alkali soils accurately in a large area. For example, hyperspectral imagery was successfully applied to estimate salt content with a model constructed from a soil salinity spectral index [23]. The HyMap imagery was used to detect and map irrigation-induced salinity with methods of matched filtering and mixture-tuned matched filtering [14].

As a result of the computational difficulties of the large data size and high spectral collinearity among the narrow bands, the estimation of saline-alkali soils using all hyperspectral bands is not productive and often results in spurious results due to noises introduced from highly correlated spectral bands. The partial least squares regression (PLSR) model was developed by Wold [24] in the late 1960s for econometrics and then introduced as a tool to analyze data in chemical [25], ecological, [26] and spectral [27] applications. PLSR is an effective linear combination of multiple predictor variables on one or multiple dependent variables [28]. The model extracts several orthogonal factors from the original variables to explore the relationships between predictor and dependent variables [26]. Thus, the PLSR model is especially useful when the variables contain random noises. The PLSR model is also suitable for the numerous and collinear predictor variables [29,30], which often result in erratic regression coefficients. PLSR is proven an effective method to relate spectra to soil properties [31,32]. In the process of building the PLSR model, predictor (spectra) and dependent (soil properties) variables can construct optimum linear solutions without introducing excessive noise.

This study tests the feasibility of the hyperspectral mapping of soil salinity and alkalinity assisted with the PLSR model and hyperspectral imager images (HSI images) in the northern Songnen Plain, a unique black soil region in China. For this purpose, the saline-alkali and non-affected soil samples were collected, and HSI images were acquired in the study area. The objectives are as follows: (1) to explore the properties of saline-alkali soils and the corresponding sensitive spectral wavelengths; (2) to estimate soil alkalinity and salinity quantitatively with soil properties and spectral bands; (3) to map the alkalinity and salinity of saline-alkali soils.

## 2. Materials and Methods

### 2.1. Selection of Study Area

**Figure 1:**
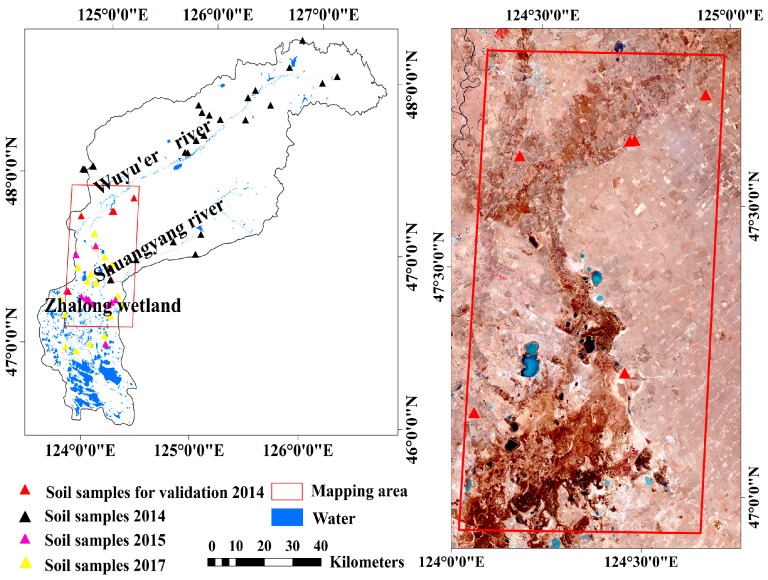
Wuyu’er–Shuangyang River Basin and soil sample sites in the study area. The red frame confines the mapping area for soil alkalinity and salinity, corresponding to two HSI scenes. The solid triangles denote soil sampling sites.

The Wuyu’er–Shuangyang River Basin in the northern Songnen Plain is selected as our study area. The basin covers an area of 23,000 km^2^ with a temperate monsoon continental climate. The annual average precipitation and temperature are ranges from 300 mm to 600 mm and approximately 3.2 °C [8]. The terrain decreases from the northeast hilly upland to the southwest low wetland. The terrain in the middle of the basin is flat, dominated by cultivated lands and grasslands. The Wuyu’er and Shuangyang Rivers are two ephemeral rivers that pass through the study area and flow into the Zhalong Wetland (Figure 1).

### 2.2. Soil Sampling and Laboratory Measurements

#### 2.2.1. Soil Sampling

Soil samples were collected along the Wuyu’er River, Shuangyang River, and Zhalong Wetland for the transition of non-affected soil and different degrees of saline-alkali soils. Figure 1 shows the sampling sites. The sampling sites were in bare soils of grasslands and cultivated lands with relatively homogeneous covers. The triangular cluster plots [33] or rectangle corner plots in an area of 100 m^2^ were applied to collect soil samples. Each triangular cluster was 30 m from the center in grasslands. Each sample point was the corner of a 20-m rectangle in cultivated lands. At each site, 3–4 samples were collected at the triangular cluster plots or rectangle corner plots. The GPS locations of all sample points were recorded. 

In total, 193 topsoil samples were collected at 63 sample sites in spring (April and May) of 2014, 2015 and 2017. All these data was divided into two datasets. Dataset 1, including 169 soil samples in 57 sample sites, was used to analyze soil properties and build the PLSR models. Dataset 2, including 24 samples in 6 sample sites collected on 26 April 2014 (close to the image acquisition dates and in the mapping area of two HSI scenes), was used to assess the accuracies of model inversion.

#### 2.2.2. Laboratory Measurements

All samples were air-dried, grounded, and filtered through 2 mm sieves [34] to remove water, large debris, stones, and stubble. Each sample of dataset 1 was split in half: one for physicochemical analysis and the other for spectral analysis. But the sample of dataset 2 was only for pH and EC measurement. In this study, pH (1:5 mixture of soil and water) [35] and EC (1:5 mixture of soil and water) [36] were measured by using a pH meter (Leici PHSJ-5, Shanghai INESA Scientific Instrument Co., Ltd., Shanghai, China) and conductivity meter (Leici DDS-307, Shanghai INESA Scientific Instrument Co., Ltd., Shanghai, China). CO_3_^2−^ and HCO_3_^−^ were evaluated by using hydrochloric acid. Similarly, these two ions were measured in an extracted solution at a 1:5 mixture of soil and water. Moreover, total organic carbon (TOC) of soil was measured with a TOC analyzer (Analytik Jena multi N/C 2100S, Analytik Jena AG, Jena, Germany). 

Spectral reflectance of soil samples was acquired by using a field portable spectrometer (SVC HR-1024i, Spectra Vista Corporation, Poughkeepsie, NY, USA). The spectrometer has a wavelength ranging from 350 nm to 1000 nm and the highest spectral resolution of 3.5 nm, matching the bandwidth of the hyperspectral image used in this study. In order to reduce spectral noises from soil water, roughness and debris, the spectra were measured using the air-dried, grounded, and sieved soil samples in a dish of 7.5 cm diameter and 2 cm depth. The measurements were collected with natural sunlight at 10:00 a.m. to 14:00 p.m. in clear days. Spectral signatures were collected at a height of 10 cm above soil surface at a nadir position, giving a 25° field of view (FOV). Before each measurement, the reflectance of white reference panel was recorded. Five reflectance spectra of each sample were averaged, smoothed, and resampled into the bandwidth of the hyperspectral image used in the present study. 

### 2.3. Hyperspectral Imagery and Preprocessing

The HJ-1A, a Chinese satellite launched in September 2008, is part of the small satellite constellation A/B/C that conducts large-scale, all-day, all-weather ecological, environmental, and disaster monitoring. The HSI is a hyperspectral sensor onboard the HJ-1A satellite. The HSI image covers the swath width of 50 km with a spatial resolution of 100 m, containing 115 bands in a spectral wavelength ranging from 459 nm to 956 nm. The HSI bands have an unequal bandwidth from 2.08 nm to 8.92 nm at an average of 5 nm. As a result of the stripe noises in the first 20 bands, only those from 21 to 115 (band centers: 505.89 and 951.54 nm) were used in this study. The HSI images in the study area (Figure 1) were downloaded at the China Centre for Resources Satellite Data and Application [37]. Two HSI images acquired on 21 April 2014 were used to map the soil pH and EC. The HSI images are level 2 products that have been roughly radiometrically and geometrically corrected. Here, each image was further geo-corrected using about 30 ground control points [23] selected from the 1:50,000 topographic maps. Atmospheric correction was also conducted using the fast line-of-sight atmospheric analysis of spectral hypercube (FLAASH) module [38] in the ENVI 5.1 software, which converted digital numbers to surface reflectance. In this way, the resampled soil and image spectra are quantitatively comparable. 

Other land covers, such as water and dense vegetation, can block the presence of soil surface, thereby introducing noises to soil detection in this study. Here, the normalized difference vegetation index (NDVI) from red (band 68 with a center wavelength of 660.58 nm) and NIR. (band 101 with a center wavelength of 841.17 nm) was extracted to identify these non-soil surfaces. Water and paddy can be separated from bare soils with NDVI < 0.05. Pixels with NDVI > 0.3 were assumed vegetation [23,39]. Residential pixels were identified through visual interpretation. All these non-soil land covers were masked out in this study.

### 2.4. Data Analysis

We adopted the PLSR model in the SIMCA-P software to explore the relationships between the HSI spectra and physicochemical characteristics (pH or EC) of the alkaline-saline soil. The multidimensional soil spectra contain not only the useful signals about the pH and EC but also noises due to complicated soil characteristics and data redundancy from spectral collinearity. The noisy signals decrease the model performance in regular regressions. The PLSR model could overcome these problems by projecting the spectral data into a low-dimensional space [31,32] and extracted the orthogonal predictor variables from all of the predictor variables (*X*). First a set of orthogonal predictor variables extracted from all the predictor variables is the first component, which is a linear combination of all the predictor variables to the greatest extent. Then, PLSR can extract the second component from predictor variables and improve the performance of the model. In theory, this process is not repeated until the increase of R^2^ is below 0.0975 [29]. Although a large number of components increase the R^2^ of the PLSR model, these components bring noises that result in decreased performance in validation. Normally, the number of components does not exceed 4 or 5 in practical applications [40]. Finally, the optimal PLSR model can be identified as a linear combination of predictor (*X*) and dependent (*y*) variables. In this study, the predictor variables (*X*) contain the HSI reflectance bands from 21 to 115. The dependent variable (*y*) is soil pH or EC. The PLSR regression model can be expressed as:

(1) $$y=Xa_{j}+F\;$$

where *F* is a constant, $a_{j}$ denotes the final regression coefficients of the corresponding HSI bands. Because the predictor variables contain the HSI reflectance bands from 21 to 115, *j* (*j* = 1, 2, …, *n*) indicates the total number of predictor variablesand *n* = 95 in this study.

During the process of PLSR model building, the performance of the PLSR model was evaluated with the R^2^, RMSE, and ratio of performance to inter-quartile distance (RPIQ) proposed by Bellon-Maurel et al. [41]. These equations used are:

(2) $$R^{2}=1-\sum_{i=1}^{p}{\left(Y-Y^{\prime }\right)}^{2}/\sum_{i=1}^{p}{\left(Y^{\prime }-\bar{Y}\right)}^{2}\;$$

(3) $$RMSE=\sqrt{\sum_{i=1}^{p}{\left(Y-Y^{\prime }\right)}^{2}/p}\;$$

where *Y* and *Y*′ are the measured and predicted values of soil pH or EC, and *Y* represents the average of measured values of soil pH or EC. *p* denotes the number of validation samples of model building. The optimal PLSR models were then applied to the HSI image to estimate the pH and EC across the study area. As the training soil spectra have been resampled to the same bandwidth of the HSI image, the prediction effectiveness of the models directly applied to images was also tested with RMSE (Equation (3)) by comparing the predicted values from the image pixels and PLSR models with the actual measured values of sample sites. The image pixels had the same location as the sample sites. *p* refers to the number of validation samples of model inversion.

(4) RPIQ=IQ/RMSE 

(5) $$IQ=Q_{3}-Q_{1}\;$$

The RPIQ index, in which the standard deviation is replaced by inter-quartile distance (IQ), accounts considerably better for the spread of the population than the ratio of performance to deviation [41]. *Q*_3_ and *Q*_1_ are quartiles. *Q*_1_ indicates the value below which 25% of the samples can be found; *Q*_3_ represents the value below which 75% of the samples can be found. RPIQ can evaluate the performance of the PLSR model by using thresholds defined by Bellon-Maurel et al. [41]. Specifically, RPIQ > 1.89 predicts excellent models, 0.76 < RPIQ < 1.89 predicts fair models, and RPIQ < 0.76 predicts non-reliable models [41]. 

## 3. Results

### 3.1. Statistics of Soil Physical and Chemical Properties

The soil physicochemical analysis in Table 1 provides the general information on soil saline and alkaline properties in the study area. The pH values of all samples ranged from 5.34 to 10.86, with an average of 8.43 and median of 9.48. The EC values ranged from 0.05 dS/m to 153.00 dS/m, with an average of 5.22 dS/m and a median of 0.78 dS/m. According to Song [42] and Rhoades [36], soil with a pH above 7.5 and EC above 4 dS/m is considered alkaline and saline, respectively. The high pH values in Table 1 reveal that soils are predominantly alkaline in the study area. The low median of EC and its large discrepancy with the mean indicates that saline soils are rare. Moreover, saline soils are also alkaline soils, which are typical characteristics of saline-alkali soils in the Songnen Plain.

The high contents of CO_3_^2−^ and HCO_3_^−^ in Table 1 may reveal further information on the soil saline and alkaline properties in the study area. The CO_3_^2−^ ion content was only found in samples with a pH above 8.7, which was close to the findings of Chorom and Rengasamy [43]. Owing to the alkalinity of the soil solution, the combination from Na^+^ and the two ions were NaHCO_3_ and Na_2_CO_3_ in the study area. NaHCO_3_ and Na_2_CO_3_ resulted in soil alkalinity. This result was also validated by the high correlation between pH and the two ions. The correlation coefficients are 0.87 between the pH and CO_3_^2−^ ion and 0.85 between the EC and CO_3_^2−^ ion (Table 2). Thus, Na_2_CO_3_ was the primary source of high pH level and salt content in the study area. Moreover, the relationship between EC and pH is high, reaching a correlation coefficient of 0.74 (Table 2). Thus, soil salinity was highly correlated with alkalinity in the study area. The TOC content values range from 0.25 to 5.71% with an average of 1.82% and median of 1.44% (Table 1). The relationships between the TOC content and EC and pH are negative (Table 2).

### 3.2. Quantitative Estimation of Soil pH and EC

In accordance with our previous research [16], soil reflectance was highly correlated with soil pH and EC in VIS-NIR ranges of field spectra. In this study, we tested the HSI image bands ranging from 505 nm to 956 nm.

Soil alkalinity in the study area was categorized into strongly alkaline (pH > 9.5), moderately alkaline (9.5 > pH > 8.5), slightly alkaline (8.5 > pH > 7.5), and non-alkaline (pH < 7.5) as described by Song [42]. Similarly, soil salinity was characterized into strongly saline (EC > 16 dS/m), moderately saline (16 dS/m > EC > 8 dS/m), slightly saline (8 dS/m > EC > 4 dS/m), and non-saline (EC < 4 dS/m) as illustrated by Metternicht and Zinck [44]. By combining these two soil properties, we categorized all soil samples into seven pH-EC levels (Table 3) with the averaged values of pH, EC, CO_3_^2−^, and TOC in each pH-EC level. The CO_3_^2−^ contents of soil pH below 8.5 were not calculated. In addition, the observation characteristics of soil surface and geographical background in soil sample sites were also listed in Table 3.

**Figure 2:**
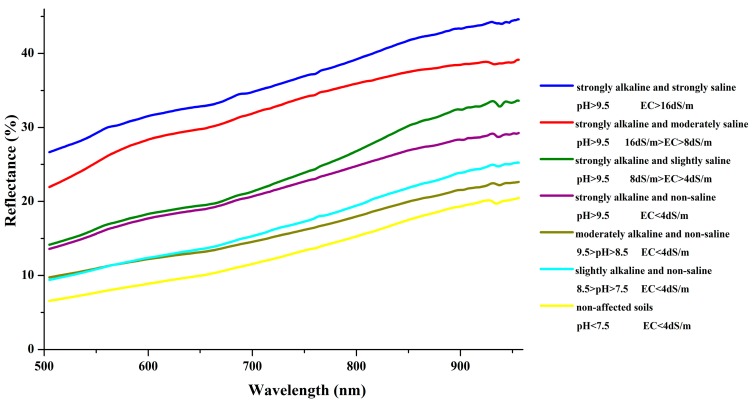
Average soil spectra curves from 505 nm to 956 nm in different pH-EC levels.

The spectra of soil samples in each pH-EC level are averaged into one spectrum and demonstrated in Figure 2. Generally, the spectral curves from 505 nm to 956 nm have similar shapes, and the reflectance decreases with low soil alkalinity and salinity levels. The trends are further significant in the 505–567 nm regions. For TOC, the reflectance decreases with soil TOC content increase except in the strongly alkaline and moderately saline level. For CO_3_^2−^, the reflectance further significantly decreases with the decrease in soil CO_3_^2−^ content. Notably, the trends are statistically significant in all levels, including the non-affected soil. So, the short wavelength bands can be used to quantitatively estimate the pH and EC in a wide range of soil salinity and alkalinity.

### 3.3. PLSR Models for Estimating Soil pH and EC with HSI-Resampled Spectra

#### 3.3.1. Prediction Performances Using HSI-Resampled Spectra

All spectra of soil samples in the range of 505–956 nm were resampled to the HSI image bandwidths (bands 21–115). The HSI-resampled spectra were used to construct the PLSR models to estimate the soil pH and EC. All the soil samples in dataset 1 were divided into different pH-EC levels and selected randomly for calibration (two-thirds) and validation (one-third). The calibration set was used to build the model and the validation set was used to evaluate the accuracy of predicted results. The PLSR models exhibit good performance when the number of components is three. The models are described as:

(6) pH=3.60+0.034b21+0.033b22+0.032b23+⋯+0.0073b114+0.0047b115 

(7) EC=−38.39+0.454b21+0.439b22+0.423b23+⋯+0.147b114+0.149b115 

where *b*21, *b*22, *b*23, …, *b*113, *b*114, and *b*115 are the spectral reflectances resampled in accordance with wavelengths of the HSI bands 21, 22, 23, …, 113, 114 and 115, respectively. The R^2^ values of PLSR models to estimate the soil pH and EC were tested at the 0.05 probability level. For pH, the PLSR model exhibits good performance with R^2^ = 0.77, RMSE = 0.95 and RPIQ = 3.84 (Table 4), suggesting a satisfactory relationship between the predicted and measured pH values. For EC, the R^2^ of the PLSR model is low (R^2^ = 0.48), the RMSE value reaches 17.92 dS/m, and RPIQ is 0.14 (Table 4), indicating the non-reliable model. When the model validation was performed, the RMSE values for pH and EC reached 1.06 and 18.92 dS/m, respectively. Nevertheless, the statistics of the PLSR models in Table 4 indicates the feasibility of the PLSR model in estimating the soil alkalinity and salinity on the basis of the resampled HSI-like spectra in the VIS-NIR region. 

#### 3.3.2. HSI-Like Band Contribution to PLSR Model

**Figure 3:**
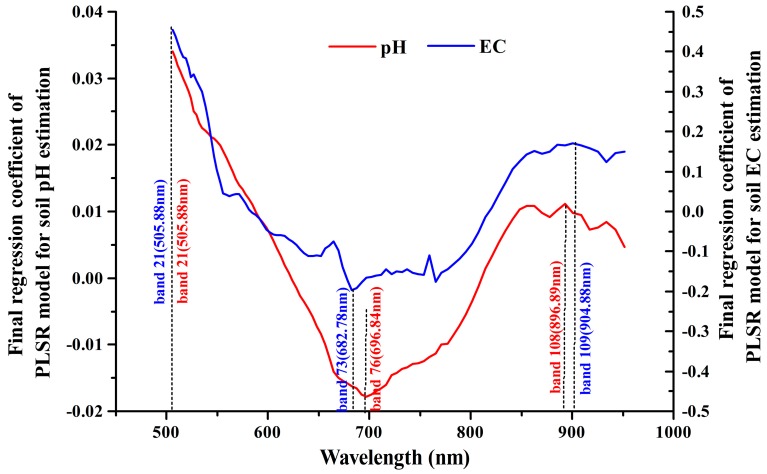
Final regression coefficient curves of PLSR models.

In the two PLSR models (pH and EC), the final regression coefficients of HSI image bands from 21 to 115 (505–956 nm) are calculated and plotted in Figure 3. The final regression coefficients can denote various contributions of these bands to the PLSR model estimation. To estimate soil pH and EC, the final regression coefficients ranged from −0.018 to 0.034 and −0.196 to 0.454, respectively. The final regression coefficient curves showed significant troughs and peaks in wavelengths which were sensitive to soil pH and EC. For the soil pH, the regression coefficient curve had two peaks and one trough that indicated the highest importance in the regression model: band 21 (band center: 505.88 nm), band 108 (band center: 896.89 nm), and band 76 (band center: 696.84 nm). To estimate the soil EC, the final regression coefficient curve had similar two peaks and one trough: band 21 (band center: 505.88 nm), band 73 (band center: 682.78 nm), and band 109 (band center: 904.88 nm). All these bands were in green, red, and NIR regions, and those at short wavelengths had high contribution to the models. As a consequence, the three sensitive bands were used instead of 95 bands to construct new PLSR models. For pH, the three-band PLSR model also exhibits good performance with R^2^ = 0.74, RMSE = 1.01, and RPIQ = 4.02. For EC, the R^2^ of the PLSR model is low (R^2^ = 0.36), the RMSE value is 19.63 dS/m, and RPIQ is 0.13 (Table 4), indicating the non-reliable model. When model validation was performed, the RMSE values for pH and EC reached 1.26 and 18.96 dS/m, respectively. The performance statistics of the three-band PLSR models were very close to those models constructed by using 95 bands. Therefore we adopt the three-band PLSR method in this study. These reflected the reasonable accuracy of PLSR method. 

#### 3.3.3. PLSR Model Inversion Using HSI Images

With the PLSR models, the soil pH and EC are derived from the bare soil pixels of HSI images. From Table 5, the maximum estimated pH and EC values were 14.65 and 35.72 dS/m, respectively. These values were reasonable and accepted. The minimum estimated pH and EC values were 1.78 and −55.09 dS/m, respectively. The low and negative values were not reasonable for models. In fact, most of these pixels are in water or wet soil area. The effectiveness of applying the PLSR models to the HSI image was tested against the measured and estimated values. Six sampling sites (marked in Figure 1) are used to assess the accuracy of the estimated soil pH and EC. All the sampling sites were in grassland and farmland near the Zhalong Wetland and rivers. In one sampling site, the ground-measured values of four samples from the triangular cluster plots were averaged to represent the value in an area of 100 m^2^. The relationship between the ground-measured values of pH and EC against corresponding image-estimated values from the PLSR models showed the accuracies of model inversion. For the pH level, the linear trend indicated a good agreement between measured and estimated values with RMSE = 1.09 (Table 5). For the EC, the linear trend indicated a low agreement between the measured and estimated values with RMSE = 17.30 (Table 5). Notably, all the image-estimated EC values were negative as a result of the non-reliable PLSR model.

**Figure 4:**
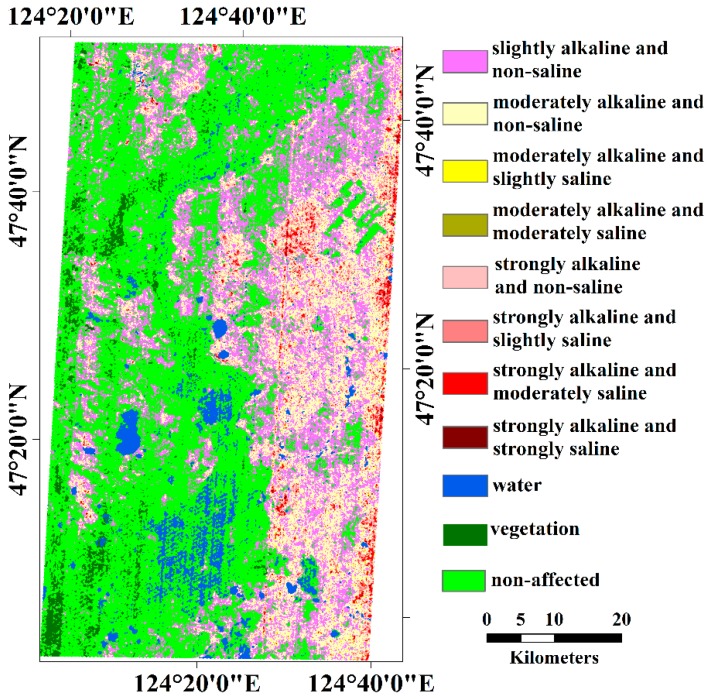
Alkaline and saline classifications of soils in accordance with pH-EC levels.

Figure 4 maps eight alkaline and saline classes of soils in accordance with the above-mentioned pH-EC levels in different color codes. Water and vegetation were separated from bare soils with NDVI < 0.05 and NDVI > 0.3, respectively. All these non-soil land covers are also color coded in Figure 4. Statistically, the alkaline and saline soils covered 46.24% of the mapping area. Slightly and moderately alkaline soils were 24.28% and 16.48% of the mapping area, which were the dominant alkaline and saline classes (88.15% of all the alkaline and saline classes), respectively. The four classes (strongly alkaline, strongly alkaline and slightly saline, strongly alkaline and moderately saline, and strongly alkaline and strongly saline) accounted for 5% of the mapping area. Notably, the two classes (moderately alkaline and slightly saline and moderately alkaline and moderately saline) were not identified in the abovementioned pH-EC levels. The two classes were negligible at 0.31% of the mapping area. In addition, the water, vegetation, and non-affected soil were 4.83%, 2.93% and 46.00%, respectively. In Figure 4, the alkaline and saline soils are near the rivers’ entrance to the mouth of the Zhalong Wetland. Compared with the distribution of alkaline soils, the saline soils were rare and overlapping on the strongly alkaline soils. 

## 4. Discussion

### 4.1. Soil Characteristics of Study Area

The Songnen Plain is located in a typical region with black soil. The physicochemical analyses of soils showed that the non-affected soil in the study area was in dark color due to the high organic matter content (TOC) in soil. As well as, the alkaline and saline soils were in low TOC content value and a light color. The soil color decreases with soil TOC content decrease and CO_3_^2−^ content increase. Variation of soil color related to the alkalinity, salinity, and organic matter content provided the potential for detecting soil alkalinity and salinity with remotely sensed data.

The analysis of soil spectra in the VIS-NIR region in this study provided a quantitative estimation of soil alkalinity and salinity. For the HSI image wavelengths (505–956 nm), high alkaline and saline soil had higher spectral reflectance than low alkaline and saline soil. This result can be attributed to the variation of soil color resulting from soil alkalinity and salinity that related to the organic matter in soil. The relationships among alkalinity, salinity, and organic matter agreed with the findings of Liu et al. [45] in saline-alkali soils in the west of the Songnen Plain, China. 

### 4.2. Spectral Sensitivity of HSI Image to Soil Alkalinity and Salinity

Normally, soil reflectance is inevitably affected by particle size, minerals, moisture, color and organic matter [15,46]. For the air-dried and sieved soil used in the PLSR model, minerals and organic matter were the primary influences. The typical zonal soils are black soil and chernozem that are characterized with high soil organic matter [47]. According to Bear [48], organic matter was one of the important influencing factors of pH and color association. The alkaline soil has high pH and low organic matter, which was in grayish color. The short VIS spectra can optimally detect the soil color and reflect the variation-related soil color more effectively than longer wavelengths [49,50]. As a result, the PLSR models based on the HSI-resampled spectra from 505 nm to 956 nm to estimate soil pH exhibited good performance in this study. Similar results are reported in other studies [34,51,52]. This case can be attributed to (1) the good correlation between the soil alkalinity and soil color; and (2) a decrease in the soil reflectance with increasing soil alkalinity in the VIS-NIR wavelengths. Compared with our previous research [16], the wavelengths of HSI images (505–956 nm) included the optimal band of OLI image for estimating soil pH. So, the PLSR models constructed with these wavelengths did well in estimating soil pH.

There were other salts contribute to soil salinity (EC) in the study area [16], which were not related to soil color and reflectance when the salts were not accumulated in soil surface. However, the wavelengths of HSI image (505–956 nm) didn’t include the optimal band of OLI image for estimating soil EC. Moreover, samples collected in new sampling sites were used to build PLSR models. High soil salinity is associated with the presence of sodium carbonate or sodium bicarbonate (i.e., maximum EC = 153.00 dS/m) in the soil of this study area, which can be represented as absorption troughs near 1930 and 2000 nm. The absence of the two absorption troughs might be attributed to the low performance of EC estimation. So, the accuracy of the PLSR model in estimating soil EC was much lower than that in estimating soil pH, as well as soil EC estimating in our previous research [16]. For these reasons, the wavelengths of HSI images cannot be better used to construct PLSR models to detect soil salinity in this study. 

Using HSI images to estimate soil alkalinity and salinity can be attributed to the sensitivity of spectral reflectance to these soil properties, especially in certain wavelengths. Past studies [53] have used the PLSR regression coefficients to approximate the strength of sensitivity for each soil factor, such as soil pH [35,51,54] and salts [27]. In this study, three HSI bands in green, red, and NIR regions had the most important contribution to soil pH and EC estimation. Green band 21 (band center: 505.88 nm), NIR band 108 (band center: 896.89 nm), and red band 76 (band center: 696.84 nm) were sensitive to pH estimation. Green band 21 (band center: 505.88 nm), red band 73 (band center: 682.78 nm), and NIR band 109 (band center: 904.88 nm) were sensitive to EC estimation. These bands were between 350–1300 nm wavelengths, which were related to the absorption and reflectance features resulted from organic matter content of black soil and saline-alkali soils [55,56] in Songnen Plain. Therefore, it is possible to estimate soil alkalinity and salinity from the HSI image.

Based on HyMap, Farifteh et al. [27] successfully constructed an artificial neural network (ANN) and PLSR with narrow bands in the range of 400–2450 nm to map soil salinity. On the basis of multispectral sensor data in the visible, near infrared and short wavelength infrared regions, multivariate adaptive regression splines (MARSs) and PLSR [50,57,58] were successfully used to map soil salinity. Thus, the linear models were suitable to describe the relationship between reflectance and soil salinity. Compared with the methods, ANN performed as well as PLSR but might take a longer time than PLSR. The PLSR model could result in an identical calibration model [27]. Some studies reported that MARS was a more suitable technique than PLSR to estimate and map soil salinity, especially in areas with high levels of salinity [58]. When satellite imagery with improved quality becomes available in future studies, we will perform a comparative analysis of the various techniques in assessing soil salinity and alkalinity in the study region.

### 4.3. Uncertainties of HSI Image Inversion

It should be noted, however, the data used in the PLSR models were narrowband in-lab spectra measured with air dried and sieved soils. While the relationships between soil spectra and salinity/alkalinity were better identified without in-situ noises from soil roughness, vegetation and moisture, etc., uncertainties were introduced when applying the models to satellite images. It was less a concern in this study because the images were acquired in a flat plain and the pixel size (100 m) is much larger than the variations of soil texture and roughness in this relatively homogeneous agricultural region. The white salt crusts (mainly carbonate) are only observed in early, dry spring before growing season starts. Moreover, crusts are rare sporadic small patches on top of soil surface. Its little impact on that of large pixel with mixed different alkaline and saline soils. In most studies of soil salinity utilizing field spectra (e.g., [15,59,60]), field spectra at multiple soil samples are often averaged to reduce the noises from field heterogeneity. Kruse [59] also found that the image spectra generally match to that of field spectra within about ±5% absolute reflectance at most wavelengths, including VIS-NIR bands. In this sense, our soil reflectance in the laboratory also reduces the field-related noise and therefore, could be applied to the 100-m HSI image in our study area. As for vegetation, it indeed can modify the spectral reflectance of salt-affected soils [61,62] and cause spectral confusion in quantifying levels of alkaline and saline soils [63]. In our study, the PLSR models included the red band both sensitive to soil pH, EC and vegetation. Careful selection of image acquisition dates could optimally reduce these vegetation effects. In late April and early May in our study area, crops are not planted and grass is just beginning to sprout. In this period, vegetation is less a concern and a threshold of NDVI < 0.3 effectively masks it out. For moisture in soils, it decreases soil spectral reflectance and, therefore, underestimates the soil salinity and alkalinity. In our study area, however, precipitation in early spring is very low [4,9], and the topsoil moisture is homogeneously low across the plain. Its effects to spectra of air-dried soil in lab were neglected in this study. The bidirectional effect of soil albedo could also affect the PLSR model development. It was not considered in this study because the HSI imagery is acquired at nadir and fixed local time. Our in-lab spectral measurement simply mimics the sun-Earth-sensor geometry of the HSI image acquisition. In future research, intensive field experiments will be conducted to further examine the above-mentioned effects to soil spectra, as well as to satellite images. Finally, in most studies of image inversion, simultaneous in-field measurements are often required to maximally match field spectra with satellite images [64,65]. It dramatically limits the usage of historical satellite data and the application in a large area, which actually are important assets of satellite remote sensing. The spectra used in this study were acquired in ideal lab conditions and eliminated the abovementioned noises. Although it introduced uncertainties in the PLSR model, by not requiring simultaneous field measurement, it allows us to examine soil properties in a large study area, or even across the basin when more ground samples and satellite imagery are added in.

Compared with the PLSR models constructed from the spectral data, the accuracies of estimating soil alkalinity and salinity were slightly lower when applied to HSI images. Thus, the PLSR model, which had three components, was an optimum linear solution. However, the stripe noises in the first 20 bands [60] still had a minimal effect on bands 21–95 and resulted in less significant strips in the inversion maps. To a certain extent, the stripe noises will bring excessive noise from HSI images and result in deviation in estimating and abstraction in classes. In addition, the PLSR models were constructed with the spectra of air-dried soils, which were different from the soil spectra in situ. Lab spectra were collected under a careful geologically orientated sampling in this study, but HSI imagery collected a far more complex spectral response in situ than the lab spectra. The water, large debris, stones and stubbles in soils can bring noises into the image reflectance and cause deviation in the model inversion from the images. Another important consideration in the deviation was the residual noises in the HSI images after atmospheric correction. In addition, the HSI images have limited bandwidths and spatial resolution. These results are also attributed to the lower quality of the HSI spectra than the laboratory-measured spectra. All these noises inevitably affected the accuracies of model inversion with HSI images.

Nevertheless, this study demonstrated a good potential to quantitatively estimate soil alkalinity and salinity with satellite hyperspectral data in the VIS-NIR regions. When better-quality hyperspectral satellites become available, the method explored in this study may be further applied to map soil properties in a large area. The optimal spectral bands identified in this study may also provide useful information for further satellite-assisted soil studies. 

### 4.4. Geographical Consideration of Affected Areas with Soil Alkalinity and Salinity

The saline-alkali soils was widely distributed in the low elevation area near the rivers’ entrance to the mouth of the Zhalong Wetland. As discussed in previous research on the Songnen Plain [4,5,6,7], this distribution characteristic of alkaline and saline soils was mainly related to the geomorphic structure. The low elevation area, such as rivers and closed-flow areas, had relatively low groundwater and could have a concentration of soluble salts (mainly sodium carbonate and sodium bicarbonate) in the basin. Thus, the groundwater in these areas was rich in soluble salts, which could accumulate on the soil surface through the movement of water and result in soil alkalinization and salinization. Being an intensely agricultural area, fertilizers will bring salts (chloride, sulfate, nitrate and phosphate), which can accumulate in low elevation areas with the river, and accelerate alkalinization and salinization or produce secondary salinization [66]. The dams can reduce water to downstream and destroy the water-salt balance. 

## 5. Conclusions

As a severe threat to the ecological environment and agricultural production, soil alkalinization and salinization have become a global concern. Therefore, detecting the soil alkalinity and salinity with a timely and spatially explicit method is necessary. This study explored the potential of assessing the soil alkalinity and salinity in the northern Songnen Plain with a hyperspectral image in a black soil region of China. Assisted with soil spectral and physicochemical analysis, the reflectance in the VIS-NIR spectral regions was further sensitive to soil pH and EC. By using PLSR models, we conducted a good prediction among soil pH, EC and HSI-like spectral bands with R^2^ values of 0.77 and 0.48, respectively. The regression coefficients of models suggested that green bands had the highest contribution to the PLSR models. Results of the HSI image inversion showed that alkaline and saline soils were distributed in the lower landscape positions, which were near the river and wetland. The soil salinity only occurred sporadically in the alkali area. The results demonstrated the usefulness of the hyperspectral imagery data, such as the HSI image to map soil alkalinity and salinity. The relatively low accuracies of HSI image inversion may come from the complicated condition of soil surface in situ and radiometric properties of satellite imagery. Distributions of HSI image-estimated soil pH and EC reflected various soil saline-alkaline properties that may be related to the intensified agricultural land use in the study area. Further investigations will be conducted when better-quality hyperspectral sensors become available. 

## Figures and Tables

**Table 1:** Descriptive statistics of soil physical and chemical measurements.

	Mean	Maximum	Minimum	Standard Deviation	Median
pH	8.43	10.86	5.34	1.91	9.48
EC (dS/m)	5.22	153.00	0.05	19.64	0.78
TOC (%)	1.82	5.71	0.25	1.40	1.44
HCO_3_^−^ (mg/L)	1247.95	4515.00	55.57	1408.92	788.14
CO_3_^2^^−^ (mg/L)	1017.96	12,436.00	0	2406.44	224.55

**Table 2:** Correlation matrix among pH, EC, TOC and two ions.

	pH	EC	TOC	HCO_3_^−^	CO_3_^2^^−^
pH	1				
EC	0.74	1			
TOC	–0.61	–0.48	1		
HCO_3_^−^	0.19	0.25	–0.07	1	
CO_3_^2^^−^	0.87	0.85	–0.58	0.16	1

**Table 3:** Descriptive statistics of soil pH-EC levels.

pH-EC Levels	Characteristics	Geographical Background	pH	EC (dS/m)	TOC (%)	CO_3_^2^^−^ (mg/L)
Strongly alkaline and strongly saline	Sporadic small patches salt crust	Margins of playas and pools	10.48	17.80	0.45	8366.70
Strongly alkaline and moderately saline	White color	Margins of playas and pools	10.46	9.42	0.57	2450.54
Strongly alkaline and slightly saline	Grey white color	Margins of playas and pools	10.29	5.64	0.53	977.08
Strongly alkaline and non-saline	Grey color	Margins of playas and pools	10.11	1.39	0.69	296.60
Moderately alkaline and non-saline	Brown color	flats near playas and pools	8.65	0.29	1.19	0
Slightly alkaline and non-saline	Dark color	flats	7.94	0.23	2.34	-
Non-affected soils	Dark color	flats	5.91	0.14	3.22	-

Note: CO_3_^2−^ contents of soil pH below 8.5 are not calculated.

**Table 4:** Performance statistics of PLSR models for estimating soil pH and EC (dS/m).

				Calibration			Validation
	Bands	R^2^	Constant	Components	RMSE	RPIQ	RMSE
pH	Band 21-band 115	0.77	3.60	3	0.95	3.84	1.06
EC	Band 21-band 115	0.48	–38.39	3	17.92	0.14	18.92
pH	Band 21, band 76, band 108	0.74	2.31	2	1.01	4.02	1.26
EC	Band 21, band 73, band 109	0.36	–55.51	1	19.63	0.13	18.96

**Table 5:** Performance statistics of PLSR model inversion from HSI images.

	Map Inversion		Validation
	Maximum	Minimum	RMSE
pH	14.65	1.78	1.09
EC (dS/m)	35.72	−55.09	17.30
